# Progress

**Published:** 1871-06

**Authors:** 


### PROGRESS.

The Missouri *Dental Journal* notices Dr. Rehwinkle's address to the late graduating class in the Ohio College of Dental Surgery, and says, "In the course of the address the Doctor makes use of the following language:

'As a specialty of medicine and surgery, operative dentistry involves a necessity for a general knowledge of the whole science of the healing art.' The world moves! The address is creditable to its author."

That the world moves, is true; but Dr. R's remark at the late commencement, is no proof of the fact; for Dr. R. never held any other sentiment, neither did the Ohio College, nor any one who was ever a member of its faculty, or in any way responsible for its teachings. The remark of the editor only indicates that the Missouri *Journal* is progressing smoothly. When one is moving without any jarring, he fancies that all he sees is in motion, just as a drunken man imagines that even the lamp posts and shade trees are staggering past him. We leave the *Journal* to draw inferences, as we have no cork screw. W.

